# An Enhanced Kaizen Event in a Sterile Processing Department of a Rural Hospital: A Case Study

**DOI:** 10.3390/ijerph17238748

**Published:** 2020-11-25

**Authors:** Valentina Nino, David Claudio, Leonardo Valladares, Sean Harris

**Affiliations:** 1 Mechanical & Industrial Engineering Department, Montana State University, Bozeman, MT 59717, USA; leovalla@gmail.com; 2 Jake Jabs College of Business & Entrepreneurship, Montana State University, Bozeman, MT 59717, USA; sean.harris1@montana.edu

**Keywords:** sterile processing department, lean healthcare, enhanced kaizen event, continuous process improvement, case study

## Abstract

Operating Rooms (ORs) generate the largest revenues and losses in a hospital. Without the prompt supply of sterile surgical trays from the Sterile Processing Department (SPD), the OR would not be able to perform surgeries to its busy schedule. Nevertheless, little emphasis has been brought in the medical literature to research on surgical instrument processing in the medical literature. The present study was done applies an Enhanced Kaizen Event (EKE) in the SPD of a rural hospital to identify sources of waste and minimize non-value-added steps in the SPD processes. The EKE consisted of three successive Plan-Do-Check-Act (PDCA) cycles, which focused on improvements at the departmental level first, then at an area level, and finally at the station level. The EKE yielded an improved streamlined workflow and a new design for the SPD layout, one of its areas, and a workstation. This paper aims at building a methodology, including identified steps. Results exhibited a 35% reduction in travel distance by the staff, eliminating non-value-added processes, reducing errors in the sterilization process, and eliminating cross-contamination for sterilized materials.

## 1. Introduction

In 2016, the United States of America (US) spent almost twice as much as other high-income countries on medical care, but utilization rates were similar to other countries [1]. The increase in US healthcare costs has promoted the pursuit of reducing waste in hospitals [2,3]. Taiichi Ohno created the Lean way of thinking about production and identified seven types of waste: inventory (stock on hand), waiting (time on hand), defects, overproduction, motion, transportation, and over-processing [4,5,6].

Lean thinking (LT) has provided good results in manufacturing industries for many decades and has been adopted in healthcare to reduce cost and waste while increasing quality and safety [7,8,9,10,11,12,13,14,15]. Some researchers argue that LT in healthcare has not proven to be as effective as expected [16]. In fact, Suárez-Barraza and Miguel-Dávila [17] recognized that there might be opportunities for improvement in the implementation and sustainability of the continuous improvement philosophy in healthcare. Researchers have studied the reasons behind this and concluded that many of the factors are centered around resistance to change due to lack of employee training and participation, lack of management involvement, lack of incentives, and lack of experience [16,17,18,19]. Nevertheless, some researchers argue that LT is well on its way to being validated in healthcare [20] and others have reported the benefits of implementing LT in healthcare [8,9,10,11,12,13,14,15,21,22,23,24]. 

Akmal et al. [24] stated the importance not just of implementing LT in healthcare, but of closing the gap between LT and Healthcare Supply Chain Management (HSCM). One example of a supply chain within a hospital is the Operating Room (OR) and all its upstream and downstream departments. ORs generate the largest revenue and losses in a hospital [2,7]. The Sterile Processing Department (SPD) is an important piece in the Operating Room Supply Chain. Without the prompt supply of sterile surgical trays from the SPD, the OR would not be able to start surgeries on-time or conduct as many surgeries in its busy schedule. Thus, it is of great importance that the SPD provides efficient and reliable instrument processing. 

Since there is great pressure to reduce waste and cost in healthcare [24], the reprocessing of reusable instruments has grown, resulting in increased workload at the SPDs [2]. Some researchers have focused on surgical tray optimization by reducing the instruments present on surgical trays or by creating trays for specific procedures [2,3,7,23,25,26]. Nevertheless, little emphasis has been brought to surgical instrument processing research in the medical literature [27]. In addition, little work has been done on implementing LT in an SPD [24,28]. 

The main idea behind LT is to identify value in any process and eliminate waste [6]. Approximately 40% of the total healthcare cost is attributed to waste [25]. Additionally, LT helps to overcome barriers between departments, encouraging them to work together, which results in better service for patients [29]. The LT tools that are most used in healthcare are 5S, Kaizen—Continuous Improvement, Value-Stream Mapping (VSM), and Visual Management [30]. 

One critical tool for rapid improvement is the Rapid Improvement Event (RIE) or Kaizen Event (KE) [5]. According to Imai [31], Kaizen, which means “continuous improvement” (CI), also refers to trying or experimenting with new ways of doing things through the Plan-Do-Check-Act (PDCA) cycle. It is a focused and structured improvement project, using a dedicated cross-functional team to improve a targeted work area, with specific goals, in an accelerated timeframe [32]. The main goal is to identify and quickly remove waste [30]. Glover et al. [22] argue that the purpose of KE is two-fold. The first purpose is to address opportunities for improvement in the process (a.k.a. the technical system). The second purpose is to address the social system by developing human resources for long-term continuous improvement. 

This article presents a case study in which a series of sequential improvement cycles within one KE was designed so that employees could learn through practice [18]. We called it an *Enhanced Kaizen Event* (EKE) as each improvement built upon the previous one through a series of PDCA cycles, and the cycles went from a macro-level to a micro-level along the improvement ramp. Many researchers have reported using an improvement ramp to slowly make enhancements to a process in which each improvement cycle is done through a different KE [21,33,34,35]. In our case, we used a different approach in which one EKE was composed of several improvement cycles along an improvement ramp; going through the improvement ramp was our EKE. The first cycle consisted of improvements at the departmental level (macro-level). The second cycle focused on improvements in an area within the department (mid-level). Finally, the third cycle made improvements at the station level (micro-level). To the best of the authors’ knowledge, this approach has not been reported in the literature before. 

The EKE was designed as a series of improvement cycles within one KE for two reasons: (1) to move several processes at (macro, mid, and micro-levels) to a more desirable and efficient state more quickly (technical system), and (2) to teach employees and hospital management about CI by example; the purpose was to create a culture of CI and reduce resistance to change (social system) [19]. Glover et al. [21] maintain that deploying KE systematically and more frequently provides conditions for success. For this reason, we decided to use the PDCA framework to add a systematic way of performing CI and conducted a series of PDCAs within one EKE to increase the frequency of improvement cycles and, therefore, increase the chances of success. The end goal was to train and empower employees so that LT and CI would be sustained after we were gone. 

This article presents both the methodology and the findings of a case study in which we developed and implemented an EKE. One of this article’s contributions is at the level of detail in the description of the continuous improvement implementation process in a sector that has undergone little research in this regard. In fact, Akmal et al. [24] recently found, that while many researchers explain why they implemented LT, only 22% out of 299 articles mentioned “how”, which speaks of the necessity for more case studies in the literature like that presented here. 

Akmal et al. [24] also found that only two articles had reported on KEs in the SPD, and only one study mentioned the seven sources of waste. This article adds to the body of knowledge by describing a success story of implementing EKE in the SPD and sharing our findings regarding the seven waste sources in our setting. It should be noted that the objective of this work, focusing on a single case, is not to generalize the conclusions but to open avenues of investigation through the conclusions that can be drawn. 

## 2. Enhanced Kaizen Event Framework

The EKE framework comes from prior work in organizational problem-solving, which is grounded in the PDCA (or Deming) cycle and the improvement ramp [33,34,36]. PDCA is one of the core elements behind the Institute for Healthcare Improvement (IHI) Quality Improvement approach (IHI-QI) [20]. It is also a core component behind the A3 problem-solving technique [20,33,34] and was designated as a Kaizen Management Philosophy Technique by Suarez-Barraza et al. [9].

**Figure 1:**
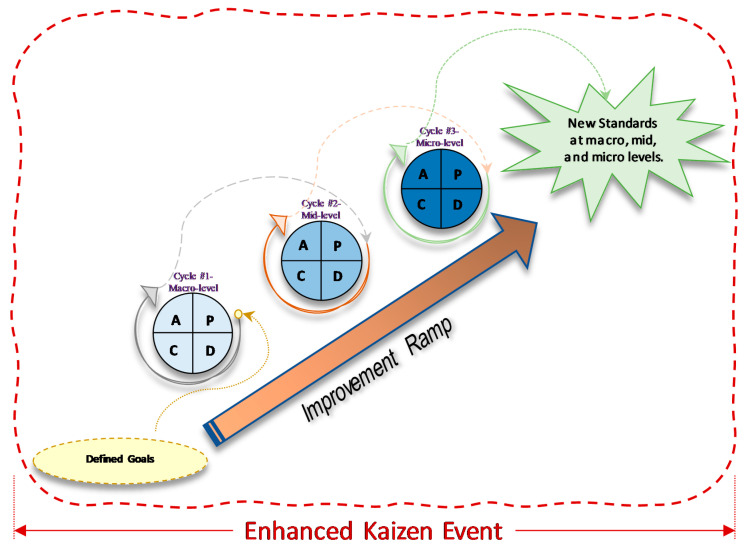
Enhanced Kaizen Event: a single KE with sequential PDCA Cycles within the KE.

The combination of successive PDCA cycles and an improvement ramp along a single KE was selected as it presents a structured way for people who have never undergone process improvement projects before. It addresses the statement of Glover et al. [21] that deploying KE in a systematic and more frequent manner provides conditions for success. It consists of a series of sequential improvement cycles within a single KE (Figure 1). Each improvement builds upon the previous one through a series of PDCA cycles along an improvement ramp. The first cycle consists of improvements at the departmental level (macro-level). The second cycle focuses on improvements in an area within the department (mid-level), whereas the third cycle focuses on the station level (micro-level).

**Figure 2:**
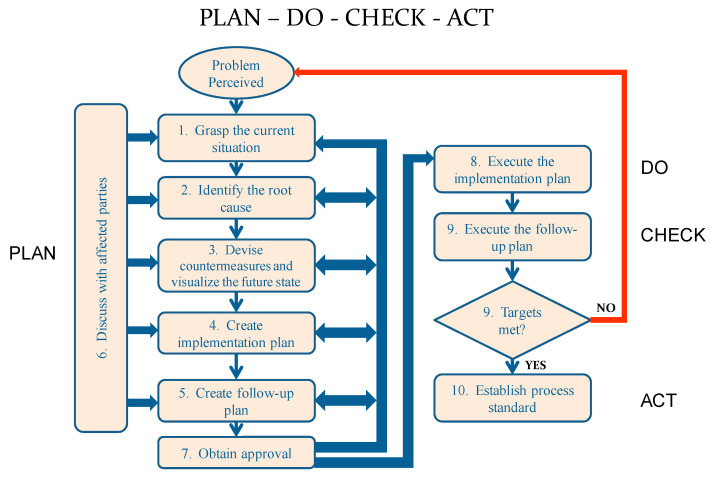
The PDCA Processes (extracted from Sobek and Smalley [34], page 20).

The PDCA cycle, presented in Figure 2, starts with grasping the current situation, which includes understanding the current process in detail, defining objectives, identifying all stakeholders, and creating a Kaizen Team (KT) composed of representatives from each stakeholder group.

The Planning stage for cycle #1 typically takes the longest time during the entire improvement ramp. A team of observers, preferably from outside of the department or the organization, needs to interview personnel, understand the current state of the system, map the flow of people, material, and information, and develop visual tools to convey the findings to the KT. The next step is to conduct root cause analysis, followed by generating ideas that address the root cause(s), in order to achieve the desired objective. Implementation and follow-up plans are then created to realize the proposed changes and validate the improvement. Throughout these steps, discussions within the KT and stakeholders occur to solicit input and agreement on the nature of the problem and root cause(s). Stakeholders’ opinions and ideas are taken into consideration to verify if the proposed course of action is the best one for the organization. These discussions may return to an earlier step to gather more data or otherwise modify the proposed plan [33].

Once an agreement has been achieved throughout the entire Planning stage of the first cycle, the “Do-Check-Act” and subsequent PDCA cycles along the improvement ramp typically occur to a short, fast-paced time-scale (the EKE). It is important to note that stakeholders’ involvement within each cycle throughout the improvement ramp is crucial to the sustainability of the new standard. In the proposed EKE, the first PDCA cycle occurs at the departmental level (macro-level), the second cycle occurs at an area within the department (mid-level), and the third cycle corresponds to the station level (micro-level).

## 3. Materials and Methods

This case study was performed at the SPD of a medium-size local hospital. The hospital is an Acute Care facility, a medium volume, 86 bed facility, with over 2000 employees of which 200 are physicians. The health professionals cover over 35 specialties and compose more than 20 clinics. SPD supports seven existing surgical suites (six surgery and one procedure room), Labor and Delivery, Emergency Department, Diagnostic & Treatment areas, patient beds, and associated on-site clinical programs. It also provides processing for the regional veterinary clinics.

SPD stakeholders in the partner hospital recognized that the department had some opportunities for improvement; SPD running behind schedule, OR delays due to late instruments, and un-sterilized trays sent to the ORs were identified as the primary concerns (problem definition). Un-sterilized trays represent a safety hazard that could cause infections or death. It has been reported that 50,000 to 100,000 lives are lost each year due to medical errors in the US alone [5]. The proposed EKE was conducted to identify sources of waste, their root causes, and countermeasures to eliminate or reduce them at the departmental level, area level, and workstation level.

KEs are generally performed in a period of three to five days and use low-cost problem-solving tools to propose and implement improvements [37]. Since healthcare delivery systems present different challenges than the manufacturing industry, Culcuoglu et al. [37] developed a novel approach to apply KEs in healthcare. They proposed two to four-hour sessions to overcome managerial problems and estimated that 8 to 16 sessions would be needed to fully execute Kaizen activities. Considering this approach and trying to avoid managerial problems, we proposed a modified Kaizen alternative that fitted better. It was decided to divide the EKE into thirty sessions of one hour to minimize interference with normal operations and reduce scheduling conflicts among the team members. We then had one session of six hours to simulate and implement the proposed solutions.

The Kaizen Team (KT) consisted of six SPD staff members (out of 12), the SPD supervisor, the OR nurse manager, one quality improvement manager from the hospital’s Quality Department, and an engineering team consisting of two industrial and management systems engineering (IMSE) graduate students, and an IMSE professor.

During the “grasping the current situation” session for the first cycle (macro-level), information regarding the current state of the process was gathered by naturalistic observations [38] and interviews [39] with the KT members and the SPD staff. With the information gathered, flow charts, a Current State Value Stream Map (VSM), and spaghetti diagrams were created to summarize and visualize the current process [40] and help identify sources of waste. Spaghetti diagrams uncover inefficient layouts and discover large distances traveled between key steps [41]. These tools allowed the KT to visualize the operation flow in the current layout.

The KT, supported by the engineering team, identified the seven types of waste in the process. The engineering team also trained the KT on how sources of waste insert variability in a process and, therefore, irregularities, unpredictability, and inefficiencies. Brainstorming [42] was used to evaluate the current state and generate new alternatives. The brainstorming sections occurred first within the engineering team and then within the KT. After these sessions, a new layout that grouped processes by areas was proposed.

The new layout was implemented (Do-Check-Act stages of cycle#1) during a six-hour EKE session on a Saturday as there were no surgeries planned on that weekend. The entire KT participated in the EKE. Metrics such as movement, number of steps, and number of times dirty and clean instruments path crossed each other were collected to assess the proposed layout. The KT then went through a second improvement cycle during the six-hour EKE. The team proposed improvements at a specific area (Prep and Pack) as part of the PDCA cycle #2. The SPD staff performed their activities for about 30 min to get a sense of the improved Prep and Pack area layout. Further changes were proposed at the workstation level during the simulation (PDCA cycle #3) and the SPD staff performed their activities once more for about 30 min to get a sense of the final tweaks. Consent was achieved, and the new layouts and workstation designs were adopted as the new standard.

## 4. Results

### 4.1. Grasping the Current Situation

After observing the process and interviewing the SPD staff, a clear picture of the SPD operation was obtained. The ORs are located a floor above the SPD. When a surgery is completed, OR nurses take all the trash, equipment, and instruments to a centralized location, which houses a “dirty elevator.” OR nurses place all the material in this elevator and send it downstairs to the SPD. The SPD process consists of three phases: Decontamination, Prep and Pack, and Sterile Processing.

During the Decontamination phase, non-disposable equipment, instruments and supplies from the ORs are received after each surgery via the dirty elevator. The SPD staff classifies the incoming material; they remove instruments from the trays and place any trash in a wheeled container. Trays are placed in a special cart designed to go inside a cart washing machine. The cleaning process is done in two stages. First, the staff manually removes any organic material. Then, the instruments are machine washed. The washing machine uses detergent products and steam to decontaminate the instruments. Although the instruments are exposed to several cycles designed to sterilize them, they are not presumed to be sterile because of the high microbial contamination present before the washing. Once the washing machine completes its cycles, the instruments are removed and set on a specific area to dry [43].

The Prep and Pack phase is the process of organizing the instruments according to recipe cards. Each recipe card has detailed instructions on how a tray should be assembled. It has a complete list of all the instruments that are included in each tray. The SPD staff assembles a tray according to an Earliest Due Date policy. They then walk to the area where the instruments are cooling and select the instruments. Each SPD technician has a workstation to perform the assembly process. Once a tray is assembled, it needs to be packaged in a way that will maintain its sterile condition until use. The materials used for the packaging must allow the sterilant to process the instruments during the sterilization process and protect the tray from contamination before it is used [43].

**Figure 3:**
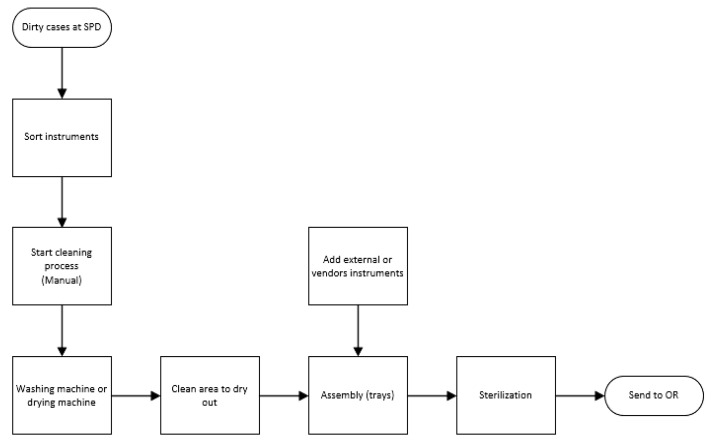
Sterile Processing Department (SPD) process flow chart.

Once a tray is packed, the SPD technician takes it to the Sterile Processing machine. The SPD at this hospital has two steam sterilizers and three low-temperature hydrogen peroxide sterilizers. The steam sterilizers run more than 90% of the workload, and the low-temperature sterilizers are used to process small trays. The trays are collected on a special cart that goes into the steam sterilizer. This cart is set in front of the machine until it is full. The sterilization process is based on pressured steam. The steam must penetrate every fiber and get in contact with any surface. Direct saturated steam is the basis of this process [43]. After the sterilization process finishes, the cart is removed from the machine and set aside to cool down. The trays are then sent up to the OR to be stored until the next surgery via a “clean elevator.” Figure 3 depicts the flowchart of the SPD process described above.

**Figure 4:**
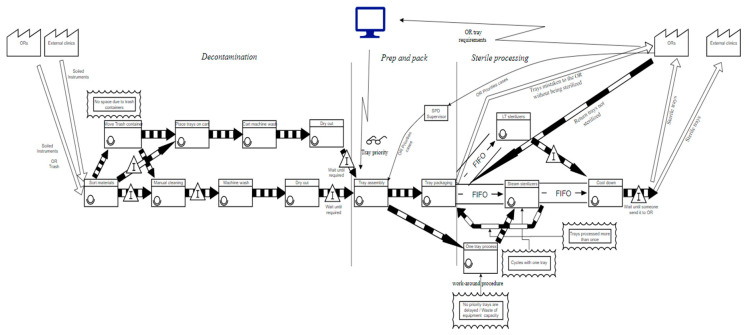
Current State Value-Stream Mapping (VSM) of the SPD.

After the team had studied the current process, it was decided to study the movement of information, materials, and people in the department to identify sources of waste. A current state value stream map (VSM) was developed to understand the SPD process’s flow of materials and information. Figure 4 presents the current state VSM. The VSM revealed that there was a lot of stagnation occurring between processes. Besides, there were a few workarounds at some operations. There was also a preemptive priority process that occurred every time the OR signaled that they needed some tools immediately.

**Figure 5:**
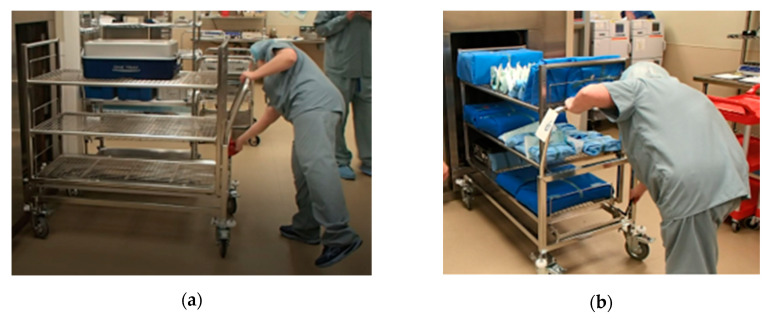
Carts going into the steam sterilizer: (**a**) Cart with one tray, (**b**) Cart at full capacity.

The team had an opportunity to observe a work-around procedure that the SPD staff followed every time they felt the instruments needed by an OR would not be ready on time. In these situations, they placed one tray on the steam sterilizer and ran the sterilization cycle with just that one tray instead of waiting for the rack to fill up and run the cycle. Figure 5 shows the difference between a full cart versus a cart with one tray going into the steam sterilizer. This procedure was the biggest source of waste that magnified the delay issue at the SPD and one of the main reasons that they were usually behind schedule.

**Figure 6:**
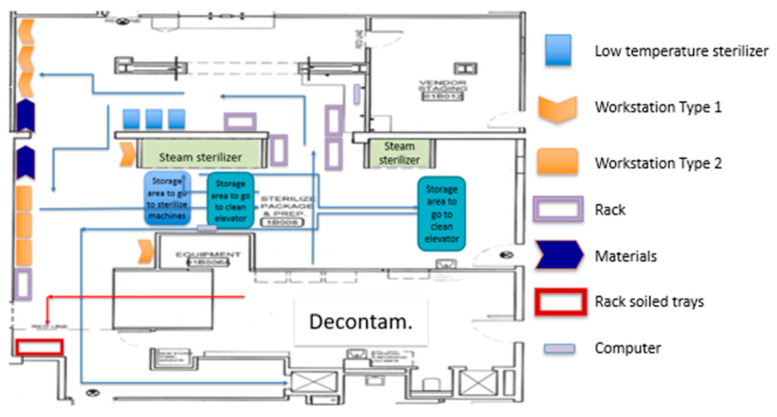
Current State SPD plant layout.

**Figure 7:**
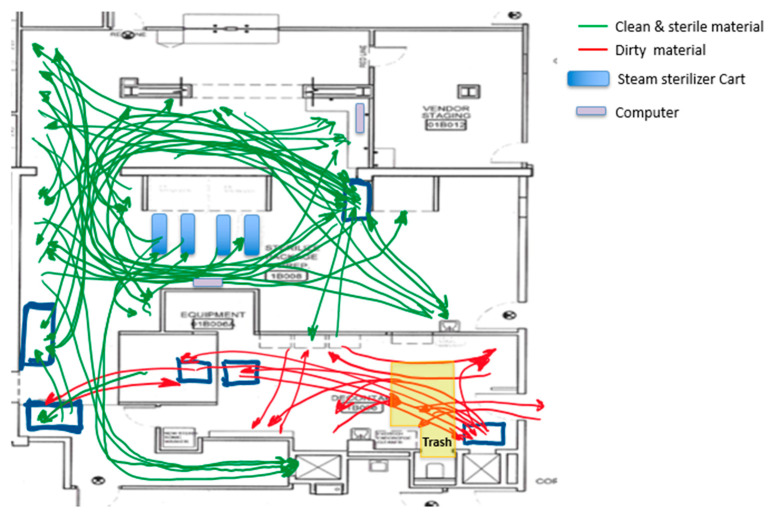
Spaghetti diagram of the current state.

A spaghetti diagram of the current state was also traced. Using this tool, we were able to appreciate the location of the equipment, workstations, storage areas, and how materials and people flowed through the SPD. This tool also allowed us to generate and evaluate new plant layouts with the KT to streamline the process flow. Figure 6 shows the original SPD layout, whereas Figure 7 presents people and material flow through the area.

From the spaghetti diagram, it was observed that the movement around the layout was erratic and messy. The staff needed to walk long distances between their workstations and the instrumentation that needed to be packed into sets.

**Figure 8:**
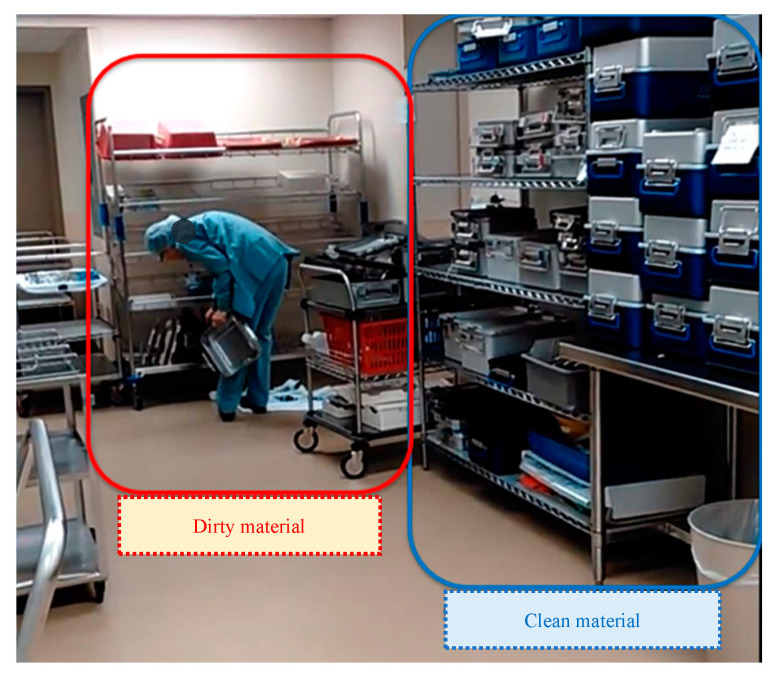
Dirty and clean materials placed in the same area.

We also observed cross-traffic between storage areas for instrumentation waiting to be sterilized, dirty trays, and instrumentation already sterilized. Part of this crossing between dirty and clean instruments can be observed in Figure 8. It was also evident that there was an overloaded area in front of both steam sterilizers that limited staff movement, reduced the use of the small steam sterilizer, and nullified the use of a computer located in that area. There were two computers at the SPD area used to check the OR surgery schedule and decide which trays needed to be assembled. Limiting the computers to just one increased the distance that some of the staff traveled and created a wait line when more than one SPD technician needed to check the OR instrument requirements.

**Figure 9:**
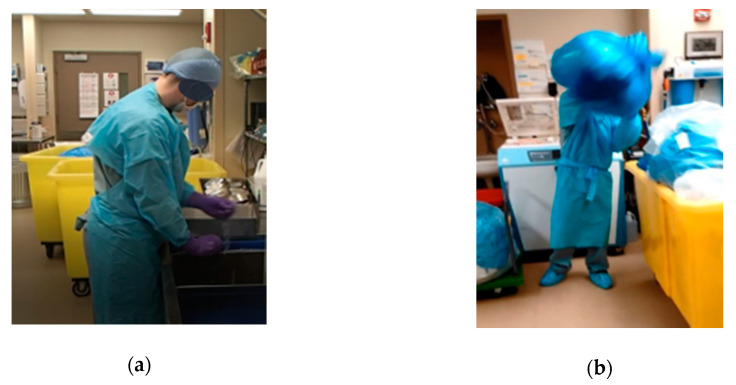
Handling of trash in the Decontamination area: (**a**) trash containers reducing workspace, (**b**) accumulation of trash.

It was also possible to identify that the Decontamination area had an issue related to lack of space due to the presence of big trash containers (Figure 9: yellow containers). Besides, it was observed that the handling of trash coming from the ORs overflowed in the area at different points in time during the day. Handling the trash at the Decontamination area caused an increase in the number of steps of the process and inefficient movement of people and materials, as seen in the yellow area in Figure 7 (Decontamination area).

One SPD technician mentioned that working in this area felt like solving a puzzle; one piece needed to be moved to create space to move the next one. Technicians needed to move the trash containers several times to be able to receive and process dirty carts and instruments.

Regarding the Prep and Pack process, we noticed that there were two different settings for workstations. The workstation type 1 (WST1) provided an ergonomic configuration with a chair and several bins placed close to the employee to facilitate the tasks. Workstation type 2 (WST2) was simply a clean table with no chair or racks on it. Each staff member decided where they wanted to work and performed all their activities related to assembly and package in the selected area.

Through the issues exposed during the observation of the current process, the KT decided to identify specific examples of the seven types of waste in the SPD process. The most important were:

- Inventory: express sterilizing equipment acquired to solve some of the SPD problems, increasing the number of available sterilizers, some of them being underused. Also, there were trays ready to go to the OR waiting on the SPD because no one was aware of them.
- Waiting: surgeries that were delayed because one or more trays were not available. Trays ready to be sterilized waiting, because the steam sterilizer was busy; this issue was magnified when the steam sterilizer was used to process only one tray. Trash waiting at the decontaminated area to be picked up and SPD technicians waiting for computer availability.
- Motion/movement: movement of trash containers in and out of the area. Movement between workstations and other areas to pick up instruments. Reaching for tools or instruments that were far out of reach of the worker. Movement to the only accessible computer.
- Transportation/transfer: long distances traveled by instruments or trays throughout the process. The need to process trays outside the decontaminated area due to lack of space. Unsterilized trays sent to the OR, ignoring the fact that unsterilized instruments cannot be taken to the OR.
- Rework/defects: trays returned to the SPD because they had been mistakenly brought to the OR without being sterilized. Trays that were rushed to the OR and skipped inspection and were later returned because they did not have all the required instruments.
- Over-processing: trays that were sterilized more than once due to disorganization and confusion on the carts in front of the steam sterilizer; an SPD staff would place a cart that had finished the cycle in front of the steam sterilizer to cool down, another technician, not aware of this, placed trays that were not sterilized and re-introduced the cart to the sterilization process again.
- Overproduction: the team did not identify any waste of this type; the process regularly runs behind schedule. SPD staff follow a list of OR needs to work on trays needed with priority.
- Waste of people’s potential (8th source of waste): before the EKE, SPD staff had never been consulted concerning the design of the workstations or the SPD layout.

### 4.2. Enhanced Kaizen Event: Going through the Process Improvement Ramp in one Kaizen Event

Given the analysis of the current state, the spaghetti diagram, and the identification of types of waste, the team did a root cause analysis using the “Five Whys” technique [34]. Five Whys is a practice of asking five times why a problem or type of waste exists with the goal of getting to the root cause of a problem. While using this technique in most of the issues and types of waste identified in the current state, it was revealed that there were major flaws in the SPD layout; the KT decided to focus on improving the SPD layout for the first PDCA cycle (macro-level).

Focusing on the layout allowed the KT to generate and evaluate alternatives that could streamline the process flow to eliminate the cross-traffic between dirty, clean, and sterilized material. The layout modification’s first objective was to separate the floorplan based on the three different processes and, therefore, on the cleanliness of the instruments: soiled instruments (Decontamination), clean instruments (Prep and Pack), and sterilized instruments (Sterile Processing). The goal was to meet the standards expected from the SPD process to ensure patient safety.

**Figure 10:**
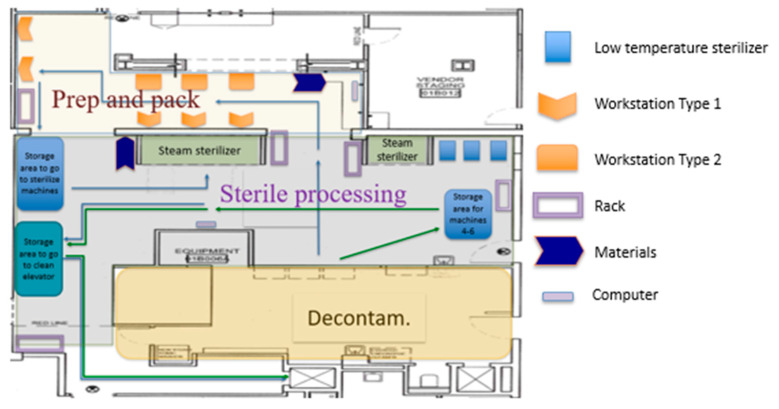
Separation of activities by phase on SPD. Proposed layout #1.

The reconfiguration of the layout aimed at reducing the types of waste identified by the team. Figure 10 exhibits the first proposed layout designed by the team. According to the first proposed layout, the low-temperature sterilizers were relocated to the Sterile Processing area. With this relocation, all the workstations were situated in the Prep and Pack area.

Another root cause the KT examined was the trash containers, which took up space and added unnecessary movement. Two KT members (the OR nurse manager and an engineering graduate student) studied the floor plans and the process that occurred at the OR prior to sending instruments and trash to the SPD through the dirty elevator. They discovered that there was no particular reason why trash needed to be sent to the SPD. It was just “the way it has always been done.” They then asked if the trash could be disposed of at the OR. The people in charge of collecting the trash were fascinated by this idea as it made sense and was preferred.

The KT members went to the centralized location where the dirty elevator is located and realized that there was more than enough space for the two large yellow containers. The OR nurse manager then changed the trash disposal policy; OR nurses were now responsible for disposing of their trash before sending the trays and instruments to the SPD. For the OR nurses, this policy change had no additional steps; before, they would place trash in the elevator, whereas now, they place it in the yellow containers.

With the trash from ORs no longer arriving at Decontamination, the processing of the trays was performed entirely at Decontamination. Due to this improvement and the movement of the workstations, space was cleared to create one area to store the trays going to the steam machine, and another area was defined to store the trays that are cooling down after the sterilization process (waiting to go to the OR). This layout improved the SPD process flow, reduced distance walked, separated instruments based on their status (eliminating the cross of dirty materials with clean and sterile materials), and improved the sterilization process (eliminated error and reduced waste).

With the help of all the KT members, equipment, racks, and workstations were moved to arrange the proposed layout during a six-hour EKE session on a Saturday. All of this was part of the first PDCA cycle in which changes were made at the macro-level by changing the entire SPD layout. Nevertheless, as with many changes, the first proposed layout (Figure 10) still presented improvement opportunities.

**Figure 11:**
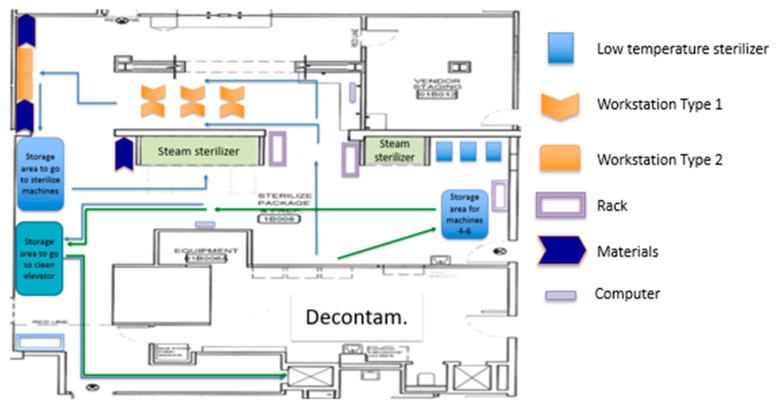
Proposed layout #2.

The engineering team led a second PDCA cycle (cycle #2), where the team focused on the Prep and Pack area (mid-level changes). The KT developed a new alternative for the Prep and Pack area. Following the team’s remark that the assembly is better performed in the WST1 and the wrapping on the WST2, the new layout aimed to improve the Prep and Pack area by executing the assembly and the wrapping process in separated workstations. Figure 11 presents the second proposed layout.

The materials needed to wrap the trays were relocated next to the WST2. Six WST1 and two WST2 were incorporated in the new design, given that, on average, the cycle time of the first process was more than three times the cycle time of the second process. Each WST1 was designed to be used by one SPD technician, and all technicians would share WST2. Due to the difference in cycle times, the second process was not expected to become a bottleneck. There were two groups of 3 WST1 in this layout. Both were facing each other. Technicians moved on corridors between the walls and the workstations. The SPD staff performed their activities for about 30 min to get a sense of the improved layout.

After the SPD staff performed their activities to test the new Prep and Pack layout and the new workstation assignments, a third PDCA cycle took place to tweak and change the workstations (micro-level). They organized the bins and materials at the stations to allow for easier reach and access. They also decided to change the orientation of the workstations. Changes to the workstations resulted in more space in the corridor and improved communication between SPD technicians.

**Figure 12:**
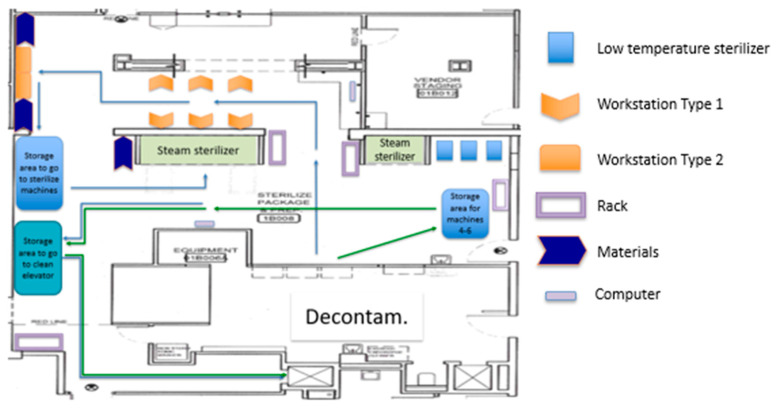
Proposed layout #3.

**Figure 13:**
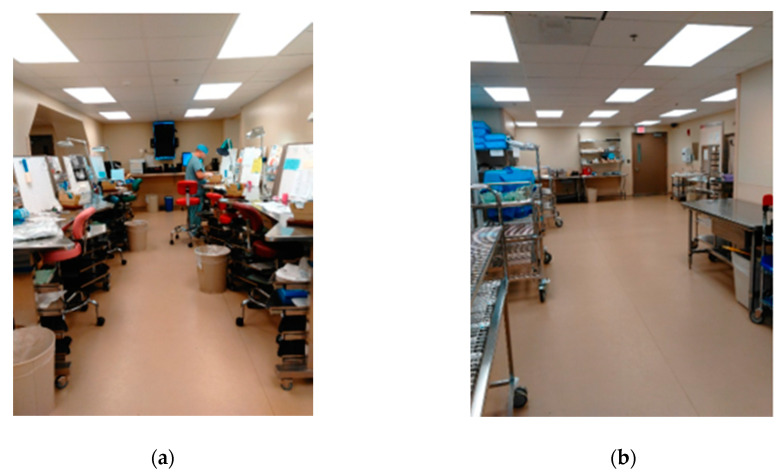
After Kaizen Event (KE): (**a**) Workstation type 1, (**b**) Space in front of the steam machines.

Other factors such as illumination, workstation design, and placement of the instruments to be assembled were addressed in cycle #3. The new configuration on the workstation improved working conditions while minimizing bending, twisting, and reaching. Figure 12 presents the third proposed layout. Figure 13 presents the new WST1 design and shows the space that has been cleared in front of the steam machines that used to be cluttered with carts related to the sterilization process.

**Figure 14:**
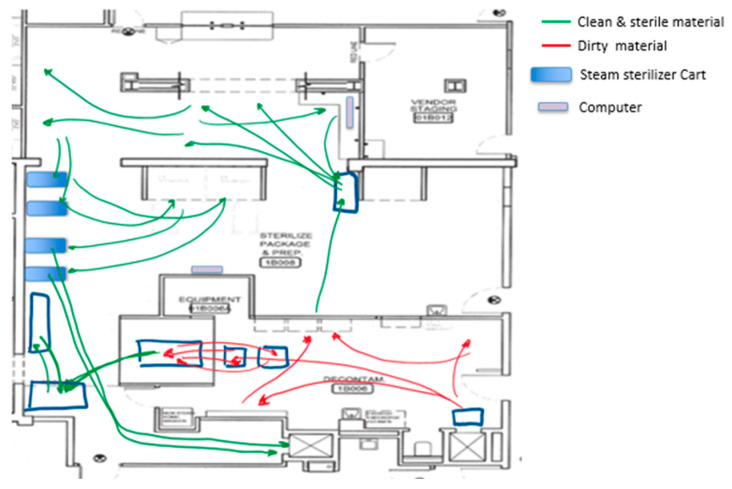
Spaghetti diagram after all the changes were implemented.

After the simulations, the KT concluded that this final layout configuration (layout #3) was a significant improvement. It provided more space in the corridor to walk and allowed the SPD technicians to communicate more easily. Once consent was achieved, all the new changes were adopted as the new standard, and modifications made during the Saturday EKE were kept in order to resume normal operations on Monday. One week later, the engineering team came back to observe the process. A new spaghetti diagram was completed to show a better understanding of the new Figure 14. presents the spaghetti diagram after the new layout was implemented. The new disposition reduced over 35% in walking distance (between workstations and equipment) and established a more streamlined workflow. Since the macro-level changes focused on designing the layout by separating the department into three major areas, cross-traffic between storage areas for dirty, clean, and sterile instrumentation was eliminated. This practice has been sustained due to the physical separation of the areas.

After the implementation of the changes, the decrease in variability was palpable. The SPD process delay decreased; rush was reduced, since processing only one tray at the steam sterilizer was no longer needed. The number of surgeries that started late due to trays not been ready was reduced by about 60%. This last improvement also helped reduce lateness in upcoming surgeries for the day.

The OR trash is not sent to the SPD anymore. Therefore, unnecessary processes have been eliminated (moving trash containers). Since this was a policy change, it has been sustained over time. The Decontamination process is faster and streamlined. Accumulation of trash at the Decontamination area increased the variability of the process during the day, given that there were less space and more trash to move every hour. Soiled trays processing used to be performed differently by different staff. One person used a cart to move trays out of the Decontamination area, and another person used to move trays one by one going in and out of the area several times.

The transfer of the trays outside Decontamination was eliminated. Since the Decontamination area now has more space, this change has also been sustained over time. This is due to the fact that the process was standardized, reducing variation; every tray follows the same process. As a consequence of the clearing of the area in front of the steam sterilizer, the second computer was available for use, reducing time waiting for an available computer and reducing variability in the process.

Given the new organization of the process, it is clear for the SPD staff which cart is being filled to go to the steam sterilizer and which cart has already finished the sterilization process. Visual management has been used to identify the status of the carts and sustain this practice. The process flow is steadier, trays that are not sterilized are not sent to the OR by mistake, and trays are not being sterilized twice. There is room for two carts in the storage area to go to the clean elevator (trays already sterilized). When a third cart arrives in this area, the first one has finished the cooling process, allowing a visual way of signaling when the trays need to go to OR (eliminating the waiting time for the finished product created by confusion about its status).

**Figure 15:**
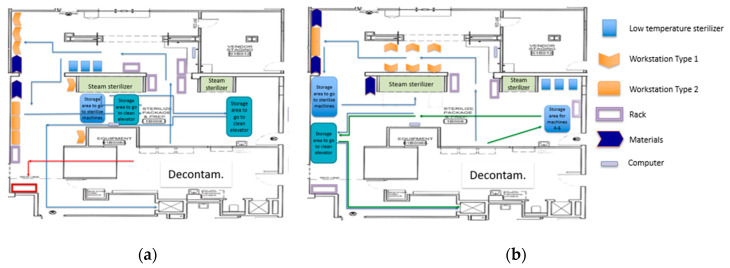
SPD layout: (**a**) Before the Enhanced KE, (**b**) After the Enhanced KE.

**Figure 16:**
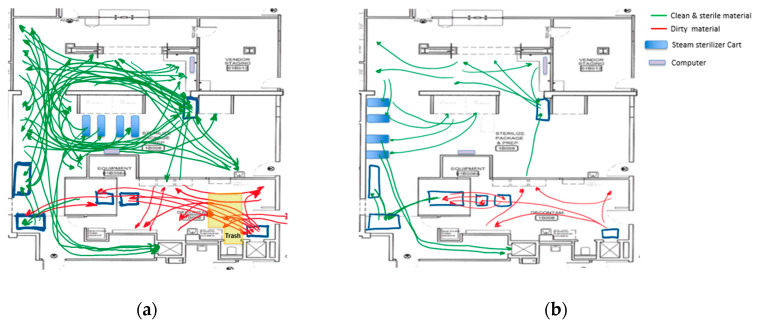
Spaghetti diagrams: (**a**) Before the Enhanced KE, (**b**) After the Enhanced KE.

Many of the changes made during the EKE have been sustained over time as they were either physical changes (layout designed by area, designated areas for carts, designated storage) or policy changes (trash containers and trash disposal). As a summary of the Enhanced KE accomplishments, Figure 15 presents before and after layouts of the SPD. In a similar manner, Figure 16 present before and after spaghetti diagrams of the flow of people and material.

## 5. Discussion

Many people believe Lean thinking (LT) to be solely about identifying and removing waste [44]. This narrow approach of LT, where most interventions are directed at reducing direct waste without understanding its implications, results in local improvements without any major effect in the overall process [44]. It could also result in transferring the sources of waste to an upstream or downstream process [24]. The underlying benefit of removing waste comes from the reduction of process variability [44]. When variability is reduced, consistency and predictability are improved, resulting in better productivity and quality [45]. Roemeling et al. [44] argue that a small effort put into knowledge dissemination on the roles of variability can have a huge impact on a Kaizen event’s success.

During this study’s execution, the engineering team invested time in educating the rest of the KT on the effects of process variability in operations and how the seven types of waste were responsible for inserting deviation from expectations into operations. We then worked as a team and identified several sources of waste and how those contributed to increased variability. By identifying specific examples of the seven types of waste (eight including the waste of people potential), the KT was able to determine assignable causes of variation that decreased SPD productivity and quality. It was conveyed that the SPD process variability was responsible for uneven arrival of trays at the ORs, causing delays in surgery start times, increasing wait times by the ORs, and, therefore, lowering OR utilizations.

The first PDCA cycle in the improvement ramp resulted in changes at the macro-level. It physically divided the SPD into physical areas, according to each process: Decontamination, Prep and Pack, and Sterile Processing. This first cycle resulted in the elimination of cross-traffic between storage areas. It also resulted in more space availability by eliminating the handling of trash at the SPD. The relocation of the trash containers was possible due to two factors: (1) including the OR nurse manager in the KT; and (2) removing steps for the SPD without adding more steps for the OR nurses. Therefore, system-wide solutions move beyond departmental boundaries to ensure waste elimination rather than waste transference to other entities [24].

During the second PDCA cycle, the KT focused on the Prep and Pack area (mid-level). It was clear to the KT that performing all the Prep and Pack processes at one workstation was not recommended, especially with two different types of workstations (WST1 and WST2) available (lack of standardization). WST1 was better suited for the assembly job since most of the disposable items were already organized and labeled in bins and drawers at the station.

The technicians who used WST2 for prep needed to walk to the nearest WST1 to collect all the disposable items needed to put in a tray to then move back to WST2. If they forgot a disposable item or grabbed the wrong one, they needed to go back to a WST1 to find the right items. Besides, all the items were clearly separated and easy to find on WST1s, in contrast to a group of mixed items in a tray that the technician needed to go through (search) each time they needed something. In fact, sometimes, a technician would spend time searching in the tray at WST2 before realizing they were missing something or had the wrong item. They would walk to a WST1 to exchange items to then walk back to their station.

**Figure 17:**
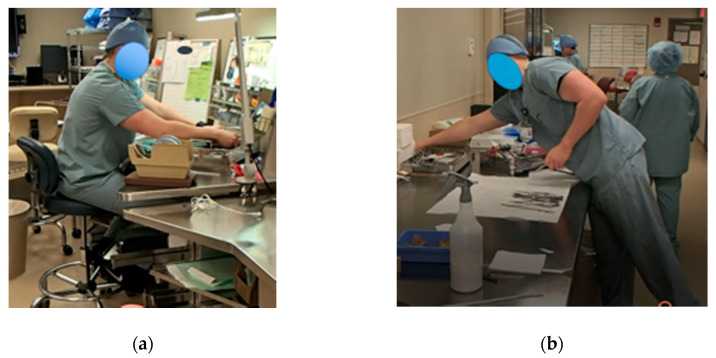
(**a**) Workstation Type 1, (**b**) and Workstation Type 2.

Figure 17 shows WST1 and WST2 before the second PDCA cycle. From the picture, two technicians doing the same task can be compared. One is comfortable sitting with all the disposable items organized within reach (WST1), and the other one has no chair and has to adopt an uncomfortable position (reaching and bending) to obtain some of the needed instruments (WST2) from a tray which is not organized or labeled.

The assembly process time varied from tray to tray, depending on the tray’s number of instruments and complexity. The average assembly time was 15.67 min, with a standard deviation of 8.00 min. On the other hand, the wrapping process time took, on average 3.99 min, with a standard deviation of 1.03 min. During this process, the tray is covered with a piece of cloth. WST2 provides more space to work for technicians since it consists of a table with nothing on it. Thus, as a result of the second PDCA cycle, it was decided to perform the assembly process on WST1 and then move to WST2 for the wrapping process.

Finally, the third PDCA cycle consisted of changes at the Prep and Pack workstation level (micro-level). Changes to the workstations resulted in more space in the corridor and improved communication between SPD technicians. Other factors such as illumination, workstation design, and placement of the instruments to be assembled were addressed in cycle #3. Improvement in working conditions was evident for the SPD staff. The new configuration of the workstation improved working conditions while minimizing bending, twisting, and reaching.

The success of the EKE was possible due to the active participation of people representing those who work in the process, the process supervisor, those who have a direct impact on the process, and those affected by the process (in our case, the OR nurse manager was in both of these groups), hospital management, and a few external people (fresh sets of eyes). Furthermore, education and training on waste elimination, CI, variability effects, workflow, and ergonomics were crucial for the adoption and solidification of this new way of thinking.

It was noticeable for the KT that small changes can greatly impact working conditions, workload, and productivity. Since the ideas came from the team, the SPD staff felt a sense of ownership and accountability. They were committed to making the changes work or further improving them. In fact, after the first PDCA cycle, the SPD technicians were constantly looking for improvement and appreciated what they could change to make a task safer, easier, and more effective. This positive change in behavior supports the LT of increasing value and decreasing waste while addressing the eighth type of waste: waste of people’s potential.

Waste of people’s potential is a hidden type of waste that people often disregard. It is addressed by empowering employees to take ownership of their processes and stations. This was an important achievement for the engineering team since we strived to create an atmosphere of CI at the SPD. Our hidden agenda was to change the way employees looked at variability, waste, and the flow of material and people. Our end goal was to teach hospital employees about LT and ignite the concept of CI in them.

To our delight, the SPD staff grasped and adopted the concept of CI. Since this EKE, they have come up with new ideas on how to further improve the process. This is evidence of how training employees and explaining the reasoning behind LT and CI can create better staff engagement. As a matter of fact, the current layout is still similar at the macro-level, but with quite a few improvements made by the SPD technicians over time at the workstation level.

**Figure 18:**
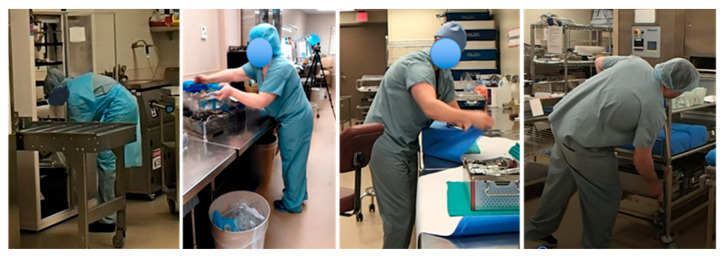
Employees adopting awkward positions before the KE.

On a final note, an additional improvement obtained by training employees on LT, waste, and variability was on the awareness of body postures, in particular when observing excessive or unnecessary motion. During the execution of this study, we observed that SPD staff were assuming awkward body postures when they were being rushed. Figure 18 shows pictures of employees assuming awkward positions before the EKE [46].

The EKE improvements allowed for a reduction in movement (stretching and reaching) and reduced the general feeling of being rushed among the SPD technicians. This is an example of the impact LT can have in ergonomics at the workstation and how an EKE can help workers identify bad work practices and find better ways to perform their activities. The improvement in ergonomics shows that EKE can have a positive long-term impact on people’s mental and physical health. The reduction in the stress level and better work practices could result in fewer work injuries and less absenteeism in the workplace.

## 6. Conclusions

This article presents both the methodology and the findings of a case study where we developed and implemented an Enhanced Kaizen Event (EKE) using the PDCA framework and the PDCA improvement ramp. The EKE consisted of three successive PDCA cycles, which focused on improvements at the departmental level first (macro-level), at an area level (mid-level), and at the station level (micro-level).

Given an analysis of the current state, the spaghetti diagram, and the identification of types of waste, the Kaizen team (KT) conducted a root cause analysis and realized there were major flaws in the current SPD layout. Focusing on the layout allowed the KT to generate and evaluate alternatives that could streamline the process flow to eliminate the cross-traffic between dirty, clean, and sterilized material.

The first PDCA cycle resulted in changes at the macro-level. It physically divided the SPD into physical areas, according to each process: Decontamination, Prep and Pack, and Sterile Processing. Layout changes resulted in the elimination of cross-traffic between storage areas. During the second PDCA cycle, the KT focused on the stations at an area level, in particular, at Prep and Pack. Six workstations were relocated to reduce the distance staff needed to walk between the workstation and the instruments that need to be packaged (more than 35% reduction).

The SPD staff performed their activities for about 30 min to get a sense of the improved layout. Changes at the station level were proposed during a third PDCA cycle, and the SPD staff performed their activities once more for 30 min to get a sense of the final tweaks. Consent was achieved, and the new layout was adopted as the new standard.

Benefits of the EKE include the elimination of cross-traffic between dirty and clean material, a streamlined process, less walking, and less searching. Besides, errors due to confusion about the status of the trays in the sterilization process were eliminated. The SPD technicians also noticed improved communication, improved satisfaction, and a positive long-term impact on people’s mental and physical health. Nevertheless, the most important benefit was on employees’ change of mindset. They now perceive every task from a CI perspective in which they constantly look for sources of waste, awkward postures, or anything that may cause a deviation from the standard or expectation (also known as sources of variability).

The three key factors that contributed towards the success and sustainability of the changes, and most importantly, the sustainability of LT and CI thinking for employees were:

(1) Identifying all stakeholders and creating a Kaizen Team (KT) composed of representatives from each stakeholder group [34]. In our case, the KT was made up of the people who work in the process, the process manager, people upstream with a direct impact in the process (suppliers), and people downstream affected by the process (customers; in our case, the OR nurse manager was in both of these categories), hospital management, and external people who can bring a fresh set of eyes. Active participation of all people is required, and all ideas are considered.
(2) Education and training on LT, CI, waste elimination, variability, workflow, ergonomics, and a holistic way of thinking [44].
(3) System-wide solutions that move beyond departmental boundaries to ensure waste elimination rather than waste transference to other entities [24].

This study illustrates how complex issues creating delays, errors, affecting hospital revenue, and threatening patients’ safety can be addressed and solved using Lean thinking (LT). EKEs can produce vast improvements in productivity and working conditions. Besides, EKEs promote the participants’ awareness of how their process can affect or improve other areas in their organization; they bring a holistic view of the processes. It should be noted that the objective of this work, having a single case, is not to generalize the conclusions but to open avenues of investigation through the conclusions that can be drawn from this case. Future research should focus on testing the proposed Enhanced Kaizen Event in different settings.
